# Explaining the climate sensitivity of junction geometry in global river networks

**DOI:** 10.1073/pnas.2211942119

**Published:** 2022-12-05

**Authors:** Callum M. Strong, Simon M. Mudd

**Affiliations:** ^a^School of GeoSciences, University of Edinburgh, EH8 9XP, Edinburgh, UK

**Keywords:** river, network, geometry, junction, climate

## Abstract

Rivers form characteristic branching patterns as they drain landscapes. Past work has shown that the angles formed between confluent channels at river junctions vary with climate aridity—but why this occurs is contested. We show how this climate sensitivity can be explained by the principle that river networks self-organize toward “optimal” configurations that minimize the hydraulic expenditure of energy. Starting from this energy minimization principle, optimal junction configurations can be calculated given three variables: the drainage area ratio of confluent channels, the scaling exponent relating channel gradient to drainage area (often called the concavity index), and the scaling exponent relating discharge to drainage area. Given that concavity and discharge–drainage area scaling vary with climate aridity, optimal junction angle theory can explain junction angle climate sensitivity. We extracted a global dataset of approximately 26 million junctions and show that the predictions of the optimal junction model are consistent with the sensitivity of junction angles to climate aridity. Our dataset includes not only the junction angle between confluent tributaries but also the “bending angles” between each tributary and the downstream channel, enabling us to quantify junction symmetry. As in the model, the geometric symmetry of real junctions is strongly controlled by the discharge ratio of the confluent channels. However, junctions with strongly asymmetric tributary drainage areas do not exhibit optimal geometries: minor tributaries show a tendency to join large rivers at the outside apex of large-scale bends.

## Significance

River networks form tree-like branching patterns as they drain landscapes. Research has shown that the angle between joining channels, referred to as the junction angle, is sensitive to climate. We develop a theory based on the principle that river networks self-organize to minimize energy expenditure along their paths and test it using the global dataset of junctions we have extracted. This theory is able to explain much of the observed variation in junction shapes, including their sensitivity to both climate and other factors, such as the drainage area ratio of the two tributaries. The theory does not, however, explain our observation that minor tributaries tend to join large rivers at the outside apex of large-scale bends.

Branching networks are common in nature. The best-known include vascular and bronchial systems, trees, dendrites, neuron arbors, and river networks. Although formed at different scales and by different processes these networks converge toward similar forms. In biology, convergent evolution indicates the presence of a shared evolutionary pressure and common function. The branching patterns of river networks have not “evolved” in a biological sense but develop spontaneously when landscape erosion is performed by flowing water (1, 2). So what unifying principles underlie the emergence of branching network patterns in nature? River networks can help us shed light on this fundamental question.

Teased from over a century of multidisciplinary work, a common thread has emerged—efficiency. The leading hypothesis is that networks grow or evolve toward configurations which optimize the efficiency of transport from any point within the network space to the inlet or outlet node (3–16). It is possible to model theoretically “optimal” networks, configured such that the cost of transport (e.g., the frictional drag of blood-flow against arterial walls, or water against the river bed) is minimized throughout (3, 5–8, 12–14, 14–17). Testing these models against observations requires metrics that encapsulate the character of network geometry. Real river networks exhibit power-law scaling characteristics with empirically quantifiable exponents, such as Hack’s Law (18, 19). The observed exponents are a close match to those expressed by locally optimal model networks (13, 20–22). While scaling law exponents are useful for characterizing networks, they do not yield precise geometric predictions about their actual shape. A network may be configured in any one of a theoretically infinite number of optimal states. On top of this basic fluidity, real networks are perturbed by the noise of the natural world and constrained by their spatial boundaries. Like fingerprints, each and every network, real or model, has a unique pattern. This makes the objective quantification and comparison of network shapes a major challenge.

One simplifying approach is to measure the geometry of network junctions. River networks can be represented as a collection of links, with junctions formed at the points where two tributary links meet and merge to form a third link, the resultant channel. The angles formed between the three links that converge at a junction are a simple metric

of local network geometry. Such angle measurements can be aggregated to statistically describe river network patterns. In this study, we apply optimal network theories to find energetically optimal junction configurations and test the results using a global dataset of 25,913,054 real junctions. We demonstrate the power of the optimal network theory to yield informative predictions about network shapes, most notably explaining how climate exerts a control on junction geometry. By improving the theoretical understanding of river network shapes we contribute to the broader study of branching network patterns in nature.

## What Controls the Geometry of River Network Junctions?

**Fig. 1.**
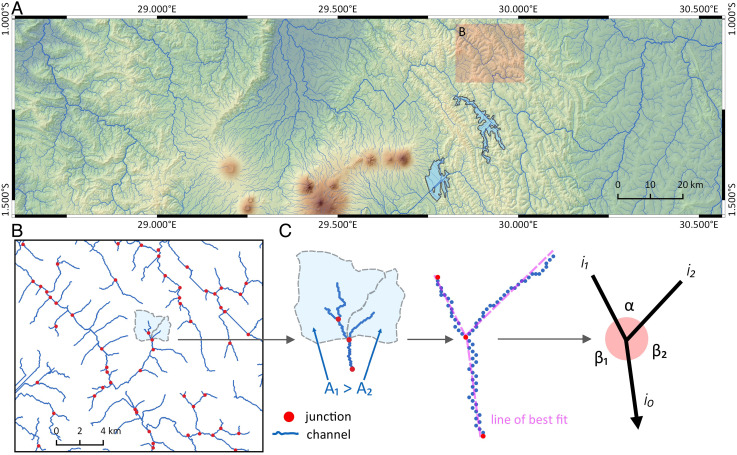
A global dataset of junctions was extracted from SRTM-DEM 30 m topographic data using LSDTopoTools (LSDTT), an open-source topographic analysis software. (*A*) An example LSDTT channel network, draped on a visualization of the SRTM-DEM data and generated at the study drainage area threshold value of 1,000 pixels (∼0.9 km^2^). The region displayed is in East Africa, showing the western branch of the East African Rift system and the volcanic Virunga Mountains. Note flow routing artifacts over lake polygons from the Global Lakes and Waterbodies Dataset where extracted channel gradients are negligible (39). (*B*) The extracted channel network at small-scale showing dataset junctions. Segments that are very short could lead to unreliable link bearings, so as a balance between completeness and reliability of bearing measurements, we eliminate junctions where any one of the three segments making up the junction is shorter than eight pixels. See *SI Appendix* for more detailed examples of junction extraction. (*C*) Schematic illustration of the measurement procedures and nomenclature for drainage areas (*A*_1_, *A*_2_), junction angle (*α*), bending angles (*β*_1_, *β*_2_), and channel links (*i*_1_, *i*_2_, and *i*_2_).

Junction angles have been intensely studied (14, 23–38) and are typically quantified by fitting a line to network links and recording the angles formed between the resulting vectors (Fig. 1), as in this study. Angles measured in this way are representative at the scale of network branching as opposed to the scale of local confluence morphology.

Any junction formed by the confluence of two tributaries has three angles. What most researchers have called the “junction angle” (*α*) is formed between the two confluent tributary channels. The other two angles are formed between each tributary channel and the resultant channel (Fig. 1). We follow the convention set by Yukawa et al. (33) in referring to these latter two angles as the “bending angles” (*β*_1_ and *β*_2_).

There has been a historical tendency to study only the junction angle (24, 29–32, 34–38). Often overlooked, the bending angles are also a fundamental measure of river network geometry as they control the direction taken by the resultant channel after it is formed at a junction. Furthermore, theoretical work (27) suggests that the junction angle may be sensitive to the ratio of the bending angles.

Several different models have been proposed to explain the shape of river network junctions. Horton (23) observed that steeper tributaries tended to be more perpendicular in their orientation relative to the main channel. He proposed a simple geometric model relating the junction angle to the slope ratio (*S*_*R*_) of the confluent channels (23, 24), where the slope of the steeper (smaller) channel is in the denominator, so *S*_*R*_ varies between 0 and 1:


[1] $$\begin{array}{c} \cos\alpha \propto S_{R}, \end{array}$$

Alternatively, several authors have advocated “optimal” models which postulate that river networks self-organize toward planform configurations that minimize total energy expenditure (14, 25–27, 30).

Another hypothesis, the “seepage” model, suggests that in humid climates, channel growth is directed by subsurface groundwater flow fields—yielding a prediction that the junction angle tends toward 72 °  (29). This model has been applied in river networks (29, 32, 35, 40) and distributary delta networks (41). Which of these models is consistent with junction angle data from real rivers?

Automated junction angle measurement has, in the last decade, resulted in the first continental-scale datasets of junction angles (34–36). Such datasets, composed of millions of junctions, are large enough to see through the considerable “noisiness” of topographic data and test hypothetical models of junction angle geometry.

Analyses of continental-scale river networks have revealed that junction angles vary systematically with climate aridity being narrower in drier climates and wider in humid climates (34–36). Junction climate sensitivity was originally interpreted in the context of the “seepage” model (35). It was suggested that a shift in the network-forming process, from groundwater seepage in humid climates to overland flow in arid climates, could be responsible for the observed climate sensitivity of junction angles (35). This finding has been used to infer palaeoclimatic conditions from ancient river networks on Mars (34, 42). It is, however, unclear how widely applicable this model is outside of river networks where seepage is the dominant network-forming process, as is the basis upon which it is assumed that overland flow results in narrower junction angles.

It was recently shown that junction angles are also correlated with *S*_*R*_ (36) as predicted by Horton’s geometric model (Eq. **1**). How can the observed correlations between junction angles, *S*_*R*_, and climate be explained analytically?

Let us start with understanding the controls on *S*_*R*_. Large rivers typically have a gentler gradient than small rivers. This relationship can be approximated by an empirical power law relating the channel gradient (*S*) to the drainage area (*A*) (43):


[2] $$\begin{array}{c} S\propto A^{-\theta }. \end{array}$$

The scaling exponent *θ* is known as the concavity index, and the *S*_*R*_ of any junction is intrinsically controlled by this exponent. When two channels with the same drainage area meet, their slopes should be equal regardless of the concavity of the network. When two channels with different drainage areas meet, their slope will be different, except where *θ* = 0. Most river profiles are concave (44), so in general, small channels are steeper than large channels. The higher the degree of concavity, the greater the contrast in the channel slope is.

Due to the topology of network branching, junctions where relatively small channels join relatively large channels are the most common and therefore dominate junction angle statistics. As such, average *S*_*R*_ reflects network concavity (36).

Network concavity is believed to be sensitive to how discharge (*Q*) scales with drainage area (36, 44). In hydrology, this scaling is represented by the exponent (*c*) from the power-law relationship between *Q* and *A*:


[3] $$\begin{array}{c} Q\propto A^{c}. \end{array}$$

Values of *c* are climate sensitive. In arid climates, *c* values are higher than those in humid climates (45), likely due to the localized nature of runoff producing rainfall events (46) and downstream water losses due to infiltration and evapotranspiration (44). Accordingly, river network concavity is correlated with climate aridity at the global scale: River profiles are more concave and have higher concavity indices in humid climates than in arid climates (36, 44).

It therefore flows logically that aridity is expressed in network concavity and, intrinsically, *S*_*R*_, due to the climatic control exerted on runoff hydrology. According to Horton’s geometric model of junction geometry, the average *S*_*R*_ then controls the average junction angle (36). This line of reasoning is supported by the observed interdependence of tributary slope ratios, network concavity, climate aridity, and junction angles (35, 36, 44).

The weakness in this logical progression is that Horton’s geometric model (Eq. **1**) is demonstrably flawed. When the two confluent channels of a junction have equal slopes (*S*_*R*_ = 1), the junction angle is predicted to be 0° (25, 27). This is clearly incorrect (24, 25, 36). A theoretical solution to this problem does exist: A modified version of Horton’s original model proves to be a special case of the optimal model of junction geometry (25, 27). In this study, we show that the line of reasoning linking junction angles, *S*_*R*_, *θ*, and climate aridity, as originally presented by Getraer and Maloof (36), becomes analytically coherent if Horton’s slope–ratio model is replaced with a model based on the optimality principle (25, 27, 47).

## Modeling Optimal Junction Geometries

Where landscapes are shaped by fluvial processes and runoff is directed down the steepest available path, local processes in river channels are predicted to drive the self-organization of river networks toward optimal configurations (1, 2). Optimal configurations are network geometries where the energetic cost (*E*) of transport through the network is minimized.

The scaling characteristics of real river networks suggest they rarely obtain perfect optimal geometries but instead tend toward locally feasible optimality: a set of dynamically accessible minimum energy states (22). River networks may modify their planform geometry by lateral channel migration or drainage capture to obtain local optimality (14, 48–50), so we might expect junction geometries in rivers to be optimal configurations (22).

How then, in practice, can we apply energy minimization principles to model optimal junction geometries? First, it is necessary to define the energy expenditure that optimization should target and minimize.

In real rivers, energy is expended through friction at the channel boundaries. Frictional energy expenditure is classically taken as the target of optimization in rivers (10, 30, 51) and can be formulated as either bed shear stress or unit stream power. A number of authors have shown that either formulation can be simplified using hydraulic geometry relationships to give a general equation for the energy expenditure (*E*) of any link (*i*), with length *L*, in a channel network (10, 13, 51):


[4] $$\begin{array}{c} E_{i}\propto L_{i}Q_{i}S_{i}. \end{array}$$

Other targets for minimization can be chosen, for example, total channel volume, but all simplify with hydraulic geometry relationships to match the form of Eq. **4** (27). The scaling laws relating slope and discharge to drainage area (Eqs. **2** and **3**) can be used to further simplify Eq. **4** to


[5] $$\begin{array}{c} E_{i}\propto L_{i}A_{i}^{\gamma }, \end{array}$$

where the energy scaling exponent, *γ*, is related to the discharge–area and slope–area and scaling exponents (*c* and *θ*, respectively):


[6] $$\begin{array}{c} \gamma =c-\theta . \end{array}$$

At a junction where two links (*i*_1_ and *i*_2_) join to form a resultant link (*i*_0_), the drainage area is additive according to a simple bifurcation rule:


[7] $$\begin{array}{c} A_{0}=A_{1}+A_{2}. \end{array}$$

In real rivers, *A* increases modestly from the upstream end of a link to the downstream end as areas proximal to the link drain into it. For simplicity, we disregard these local increases in *A* and assume the *A* of any junction link conforms to Eq. **7**. The drainage area ratio (*A*_*R*_) of a junction is given by


[8] $$\begin{array}{c} A_{R}=\frac{A_{2}}{A_{1}}\;\;\text{where}\;A_{1}\geq A_{2}. \end{array}$$

The total energy expenditure, *E*_*total*_, of a network junction can be calculated by summing the *E* (given by Eq. **5**) of each link (30):


[9] $$\begin{array}{c} E_{\mathrm{total}}\propto L_{1}A_{1}^{\gamma }+L_{2}A_{2}^{\gamma }+L_{0}A_{0}^{\gamma }. \end{array}$$

A junction’s configuration is optimal when *E* is at a minimum.

Finding the optimal configuration of a junction now becomes a mathematical problem. To visualize this problem, imagine three points of a theoretical junction are fixed: the starting points of *i*_1_ and *i*_2_ and the ending point of *i*_0_. These three points define the spatial domain of our hypothetical junction. The angles formed between the channel links effectively define the position of a fourth point, the junction. The angles must now be chosen so as to position the junction in the location that minimizes the function of *E*_*total*_ (Eq. **9**). The problem is in effect one of length minimization where the junction link lengths are weighted according to *A*_*i*_^*γ*^. This proves to be a version of the Fermat–Weber problem, well known in location theory (52, 53). A solution to this problem (developed in the context of arterial branching) relates the weighting factors of the links (in our case *A*_*i*_^*γ*^) to the optimal junction angle and optimal bending angles (47):


[10] $$\begin{array}{cc} \cos\alpha & =\frac{A_{0}^{2\gamma }-A_{1}^{2\gamma }-A_{2}^{2\gamma }}{2A_{1}^{\gamma }A_{2}^{\gamma }}. \end{array}$$

[11] $$\begin{array}{cc} \cos\left(\beta_{1}\right) & =\frac{A_{2}^{2\gamma }-A_{0}^{2\gamma }-A_{1}^{2\gamma }}{2A_{0}^{\gamma }A_{1}^{\gamma }}. \end{array}$$

[12] $$\begin{array}{cc} \cos\left(\beta_{2}\right) & =\frac{A_{1}^{2\gamma }-A_{0}^{2\gamma }-A_{2}^{2\gamma }}{2A_{0}^{\gamma }A_{2}^{\gamma }}. \end{array}$$

The reader is referred to Zamir (47) for the full derivation of these equations which involves, in their words, “much tedious algebra.” Using Eqs. **7** and **8**, the optimal junction Eqs. **11**, **12**, and **10** can be simplified to (27):


[13] $$\begin{array}{cc} \cos\alpha & =\frac{{\left(1+A_{R}\right)}^{2\gamma }-1-A_{R}^{2\gamma }}{2A_{R}^{\gamma }}. \end{array}$$

[14] $$\begin{array}{cc} \cos\left(\beta_{1}\right) & =\frac{A_{R}^{2\gamma }-{\left(1+A_{R}\right)}^{2\gamma }-1}{2{\left(1+A_{R}\right)}^{\gamma }}. \end{array}$$

[15] $$\begin{array}{cc} \cos\left(\beta_{2}\right) & =\frac{1-{\left(1+A_{R}\right)}^{2\gamma }-A_{R}^{2\gamma }}{2A_{R}^{\gamma }{\left(1+A_{R}\right)}^{\gamma }}. \end{array}$$

**Fig. 2.**
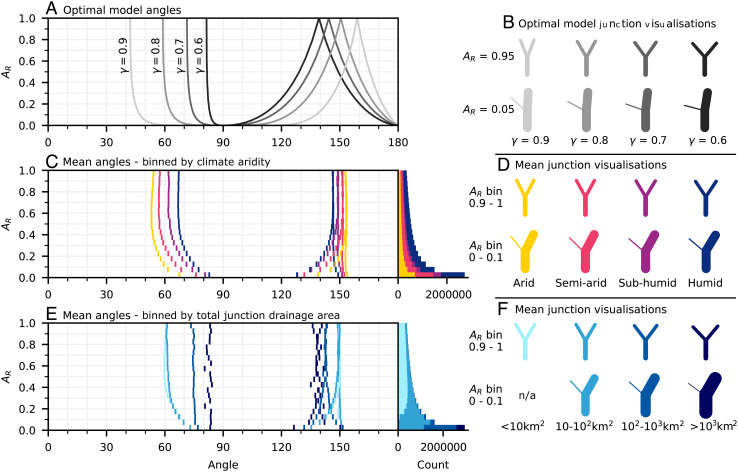
Based on a global dataset, river network junction geometry varies systematically with both climate aridity and total junction drainage area for all junction drainage area ratios. (*A*) Optimal junction geometries generated using Eqs. **13**–**15** for different drainage area ratios (*A*_*R*_) and selected values of the energy scaling exponent *γ*. See *SI Appendix* for the full range of optimal geometries. Grayscale shades represent optimal geometries found for different values of *γ* (labeled correspondingly). There are three lines in any single grayscale shade representing the junction angle and bending angles. (*B*) Visualizations of optimal junction configurations generated using Eqs. **13**–**15**. The *Upper row* of junctions illustrates optimal model behavior at a high *A*_*R*_ value (0.95) and the *Lower row* a low *A*_*R*_ value (0.05), with *γ*, as labeled, decreasing from *Left* to *Right*. Grayscale shades correspond to lines in (*A*). (*C*) Mean junction geometries binned by climate aridity zone and *A*_*R*_. The number of junctions in each data bin is shown in the histogram adjoined to the plot. For full statistics, please refer to *SI Appendix*. (*D*) Junction visualizations constructed from the mean values of angle data from *A*_*R*_ bins of 0.9 to 1 and 0 to 0.1, with aridity zones as labeled. In effect, these illustrate what the global “average” junction looks like for both junctions where the tributary drainage areas are approximately equal (*Upper row*) and junctions where the tributary drainage areas are strongly aysmmetric (*Lower Row*). (*E* and *F*) are, respectively, the same as (*C* and *D*) except with junction angle data binned according to the total junction drainage area as opposed to climate aridity. Aridity and total drainage area colours in all plots are as labeled in (*D* and *F*), respectively. The line thicknesses of the channel links in the junction visualizations plots (*B*), (*D*), and (*F*) are relatively scaled so as to represent the discharge ratio (*A*_*R*_) of confluent tributary links. The *A*_*R*_ is taken as the mean *A*_*R*_ value of the binned data in the case of the plots representing real data. The line scaling is performed according to the hydraulic geometry relationship between planform channel width (*W*) and discharge (*Q*), *W* ∝ *Q*^0.5^, with drainage area (*A*) used as a proxy for *Q*. As such, the relative widths in the figure are roughly similar to those of real rivers. Note that due to the threshold drainage area for extraction (1 km^2^), there are no junctions in the dataset with both *A*_*R*_ less than 0.1 and drainage area less than 10 km^2^.

These equations show that if junctions are in optimal configurations, their geometry should be controlled by the area ratio (*A*_*R*_) and the energy scaling exponent *γ* (Fig. 2*A*). The parameter *γ* is climate sensitive: It is calculated by subtracting the slope–area exponent from the discharge–area exponent (i.e., *γ* = *c* − *θ*; Eq. **6**). Values of *θ* are low where climate is arid and high where the climate is humid (36). Conversely, *c* values increase with increasing aridity (e.g., refs. 45 and 46). Therefore, *γ* values should be relatively high in dry climates and relatively low in humid climates (Eq. **6**).

In summary, optimal junction angle theory (27, 47) predicts that junction geometry should be climate sensitive. We now test this prediction using a global dataset of junction geometries.

## Results

To explore the geometry of real river networks, we extracted a global channel network and measured the junction geometry of 25,913,054 individual junctions. Junctions with low channel slopes were removed due to flow routing concerns, leaving 15,904,307 suitable for analysis (see *Materials and Methods* for further information). We measured *A*_*R*_ directly and recorded the ratio of mean annual precipitation to mean annual evapotranspiration, known as the “aridity index” (AI), at the location of every junction to test the theorized link between climate and the energy scaling exponent *γ* (Eq. **6**). We compared the results against theoretically optimal junction configurations generated using Eqs. **13**–**15** (Fig. 2), where *γ* was assumed to be the same value in all three links.

### Junction Angle Climate Sensitivity Is Consistent with Optimal Junction Theory.

As the energy scaling exponent (*γ*) decreases, the optimal junction angle widens and the optimal bending angles become narrow (Fig. 2*A*). Since *γ* increases with increasing aridity, we expect to see narrow junction angles in arid climates and wide junction angles in humid climates across all drainage area ratios. To demonstrate the climate sensitivity of junction geometry, we classified the junctions by their aridity index climate zones (*SI Appendix*) and binned the data for different values of *A*_*R*_ as shown in Fig. 2.

Junction angles are systematically wider in humid climates and narrower in arid climates across all values of *A*_*R*_. The spectrum of junction angles that occur across climate aridity zones can be reproduced by modeling optimal junction configurations with different values of *γ*. The observed tendency of junction angles to widen with increasing tributary drainage area ratio (*A*_*R*_) is also predicted by the optimal junction model.

### Junction Geometry Is Sensitive to the Tributary Drainage Area Ratio.

In the optimal junction model, the area ratio (*A*_*R*_) effectively controls the relative size of the bending angles and therefore junction symmetry (Fig. 2*A*). When confluent tributaries have similar drainage areas (*A*_*R*_ ≈ 1), the model predicts that junctions should be symmetrical along the axis of the resultant channel with equal bending angles, in other words, shaped like the uppercase letter “Y”. As *A*_*R*_ decreases from unity, this symmetry is predicted to break down; the junction should be shaped more like the lowercase letter “y” (see Fig. 2*B* for visualizations of this effect).

**Fig. 3.**
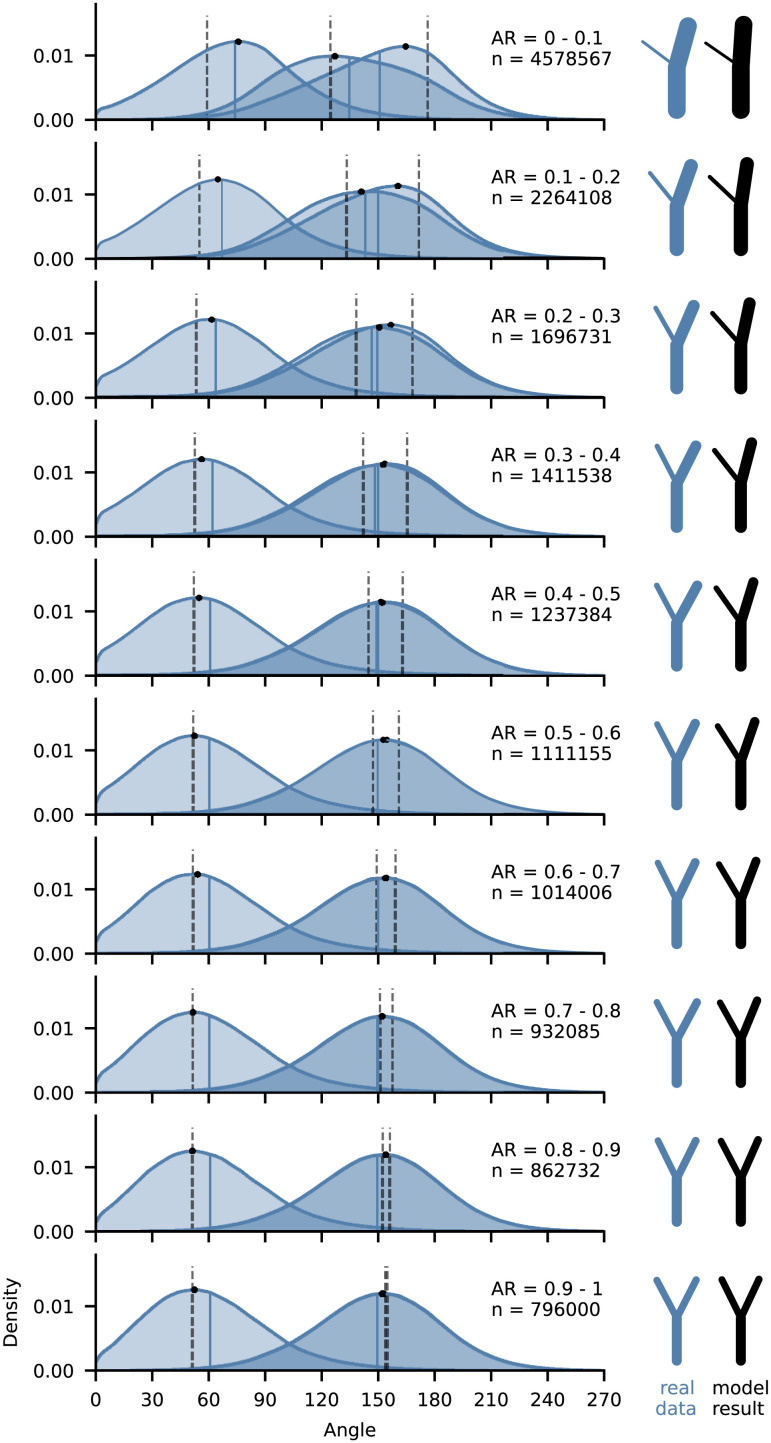
Junctions lose geometric symmetry as one tributary becomes larger than the other, as shown here by kernel density estimate (KDE) distributions of junction angles and bending angles with data binned by *A*_*R*_. Black dots are peak KDE values. Blue vertical lines are bin means. Gray vertical lines are optimal junction angle solutions calculated using mean bin *A*_*R*_ and *γ* = 0.85 (Eqs. **13**–**15**). Visualizations of the peak KDE angles and optimal junction angle solutions for mean bin *A*_*R*_ and *γ* = 0.85 are displayed on the right.

Fig. 3 shows that real junctions are, as predicted, geometrically symmetrical (“Y” shaped) when the tributaries are equally sized. When the *A*_*R*_ of the tributaries decreases below 0.5, the bending angle distributions begin to diverge and become skewed. This means that as the size of one tributary at a junction increases beyond twice that of the other, junction symmetry breaks down (that is, the junction becomes more “y” shaped).

Junction angles get wider as *A*_*R*_ decreases, as predicted by the optimal junction angle equations (Fig. 2) (subject to the condition that *γ* >  0.5, see *SI Appendix* for more discussion). The widening of the junction angle occurs at the expense of both bending angles equally when confluent tributaries are equally sized (*A*_*R*_ = 1) but predominantly at the expense of the typically smaller bending angle (*β*_2_) as one tributary becomes substantially larger than the other (i.e., as *A*_*R*_ approaches zero).

The bending angles in real junctions diverge less rapidly with increasing *A*_*R*_ than predicted by the optimal junction theory. It is only once *A*_*R*_ exceeds 0.5 that real junctions become asymmetrical, whereas in optimal model junctions this occurs from *A*_*R*_ values close to unity.

When one tributary is substantially larger than the other (i.e., where *A*_*R*_ approaches zero) the bending angle between the larger tributary and the resultant channel does not reach the optimal 180° predicted by the optimal junction equations (Figs. 2 and 3). This is associated with a greater-than-predicted increase in the junction angle when *A*_*R*_ is low.

### Junction Geometry Varies with Total Junction Drainage Area.

Our dataset shows that junction geometry varies systematically with total junction drainage area across all *A*_*R*_ values. Junction angles are relatively wider in junctions with large drainage areas (Fig. 2) than in junctions with small drainage areas. The relationship between junction geometry and total junction drainage area roughly mirrors the relationship between junction geometry and climate, although the trends in the former are more poorly resolved at large drainage areas due to the inherent scarcity of such junctions in the dataset.

If real junctions (when statistically aggregated globally) exhibit optimality—as we show to be broadly plausible—then the observed relationship between total junction drainage area and junction geometry suggests that the energy scaling exponent *γ* decreases with increasing drainage area in real rivers.

### Empirical Values of γ, the Energy Scaling Exponent.

Comparing observed junction geometry to theory allows us to empirically infer global average values of *γ* (Eq. **6**) over the studied range of climate and total junction drainage area. Based on the binned data averages as shown in Fig. 2, we see that the value of *γ* is typically between 0.6 and 0.9. For junctions where the tributaries are effectively equally sized (*A*_*R*_ ≥ 0.99), the global mean junction angle is 61° and corresponding value of *γ* is 0.79 (as calculated using Eq. **13**).

## Discussion

Our global analysis of over 15 million junctions shows that optimal junction angle theory (27) is able to capture many of the key geometric features of real river network junctions.

In our dataset, the geometry of junctions in river networks varies systematically with climate aridity (Fig. 2), thereby confirming the results of previous studies (34–36). We also observe a similar pattern in how junction geometry varies with the total junction drainage area. This is consistent with the previous observation that junction angles widen with increasing junction stream order (35). The broad alignment between optimal model predictions and the shape of real junctions supports our central hypothesis that climate-sensitive flow accumulation is expressed in network geometry as rivers self-organize toward energetically efficient configurations. However, we note substantial deviations from optimal geometries in junctions with strongly asymmetric tributary drainage areas.

### Drainage Area Ratios Exert Control on Junction Angles.

Horton (23) noticed that small steep streams joining large channels appeared to do so with relatively large junction angles. Supporting his observations, we demonstrate that, at global scales, junction angles systematically widen as one tributary becomes larger than the other (Fig. 2). This is predicted by the optimal junction model and is consistent with previous studies showing that junction angles increase as the difference in the Horton–Strahler stream order of the confluent tributaries (effectively a proxy for *A*_*R*_) increases (24, 26).

Getraer and Maloof (36) found that junction angles also vary with *S*_*R*_. This is consistent with our findings: there is a strong empirical relationship between *S* and *A* in real rivers (Eq. **2**), so we expect *S*_*R*_ and *A*_*R*_ to covary. The optimal junction angle equations can also be formulated in terms of *S*_*R*_, but using these formulations requires known or assumed *θ* values (see *SI Appendix* for further discussion).

### Linking Climate, Runoff Hydrology, and the Energy Scaling Exponent.

Typical values for the concavity index, *θ*, are ∼0.3 in arid climates and ∼0.5 in humid climates (36). Based on our fitted values of *γ* and reported values of *θ*, we can calculate the apparent value of the discharge–drainage area scaling exponent *c* using Eq. **6**. This exercise suggests the effective value of *c* is ∼1.25.

The empirical value of *c* from our calculations, while consistent with some direct measurements of *c* made using peak annual discharge (54), is notably greater than most published estimates made using mean annual discharge (45, 55–57). However, direct comparison of these values should be approached cautiously: Our *c* value infers a geomorphically effective discharge and it is not clear how this relates to direct measures of discharge (58).

Due to the existence of critical thresholds for sediment transport and channel incision, the variability of discharge may serve as a better proxy for geomorphically effective discharge than the absolute magnitude of discharge (59, 60). Runoff events are typically attenuated downstream (61–63). This occurs because with increasing spatial scale, it becomes increasingly unlikely that runoff-generating precipitation events will be evenly distributed across catchment areas in both time and space. As such, the relationship between discharge variability and drainage area becomes increasingly nonlinear with increasing basin scale (*c* decreases downstream) (46, 61–63). An illustration of this effect, and of particular pertinence to junction analysis, is that *c* values for peak discharge events instantaneously decrease after large tributary confluences (64, 65). This is consistent with our finding that the inferred energy scaling exponent, *γ*, decreases downstream as the junction drainage area increases (Fig. 2).

Discharge variability is sensitive to both climate aridity (66, 67) and basin drainage area (46, 63–65). Given the similarity of the variations in junction geometry across the range of both climate aridity and total drainage area (Fig. 2), we speculate that river network geometries are optimized in relation to a geomorphically effective discharge, linked to discharge variability through threshold effects (e.g., refs. 59 and 60).

### Why Do Small Tributaries Apparently “Deflect” Large Rivers?.

There are two predictions about junction shapes that one might make given physical intuition. One is that if the two confluent tributaries are the same size (*A*_*R*_ = 1), the resulting junction should be symmetrical (shaped like the uppercase letter “Y”). The second is that at a junction where a small river joins a much larger river (where *A*_*R*_ is close to 0), the path of the large river should be minimally deflected from its original orientation upon exiting the junction (the junction should be asymmetrical and shaped like the lowercase letter “y”). Some past authors have implicitly made these assumptions about junctions (e.g., refs. 30, 40, and 68). The optimal junction angle equations (25, 27, 47) theoretically affirm these intuitive predictions as the end-members predicted to occur at the possible limits of drainage area ratio values, 0 and 1; as *A*_*R*_ increases, the size of the optimal bending angles becomes unequal, resulting in the loss of junction symmetry (Fig. 2*A*).

Although a breakdown of geometric symmetry occurs synchronously with the breakdown of drainage area symmetry (decreasing *A*_*R*_) in real junctions (Figs. 2 and 3), it is clear from our data that junctions with strongly asymmetric drainage areas (low *A*_*R*_ values) do not exhibit locally optimal geometries. On average, larger rivers are substantially “deflected” following the confluence of much smaller tributaries. Fig. 3 shows that in junctions with over an order of magnitude difference in tributary drainage area (*A*_*R*_ between 0 and 0.1), the average bending angle formed by the larger tributary and the resultant channel is between 150° and 160°, as opposed to close to 180°, as optimally (and intuitively) predicted. So how can the observed “deflection” of large rivers by small tributaries be explained?

The complex hydrodynamics of river confluences and associated sedimentation and erosion (e.g., ref. (69) and references therein) may plausibly promote the lateral migration of network junctions in the direction of the smaller tributary, causing the larger river to form a bend, with the tributary joining at the outside apex. This explanation seems unlikely for two reasons. First, our junction angle measurements reflect the network-scale structure rather than the functional scale of confluence hydrodynamics. Second, it seems inconceivable that the paths of very large channels could be meaningfully impacted by channels orders of magnitude smaller.

What appears as the “deflection” of large rivers by minor tributaries may alternatively be interpreted as minor tributaries preferentially joining large rivers at the outside apex of large bends. In other words, the location of the junction may be influenced by the location of the bend, rather than vice-versa. This interpretation is supported by the observation that developing tributaries nucleate at the outside apex of bends when tidal channel networks form (70).

The undercutting and steepening of slopes on the outside of channel bends may encourage erosion and tributary development. Such mechanisms may influence the self-organization of minor tributaries around larger channels. Tributaries may treat larger channels, with their inherent sinuosity, as fixed boundaries. This interpretation is consistent with the basic prediction of the optimal junction angle model that larger channels should be scarcely influenced by the confluence of minor tributaries.

The optimal junction model is limited by its mathematical simplicity and locality, meaning it cannot effectively accommodate the sinuosity inherent in real rivers. These limitations may explain the poor predictive capabilities of the model at low drainage area ratios. Minor tributaries joining large rivers may reduce their total channel length by doing so at the outside apex of a large-scale bends. As such, these configurations may be energetically optimal at the network scale.

Optimal Channel Networks (OCNs) where junction geometries are free to evolve can be found computationally (14). In models driven to an overall state of dynamic optimality, junctions with strongly asymmetric drainage areas (low *A*_*R*_) exhibit deviations from optimal geometries very similar to those we observe in real networks (14). This suggests that our observations should not be interpreted as an invalidation of the optimality theory. Instead, our results suggest that optimality at the network scale may require the sacrifice of local geometric optimality for junctions with low *A*_*R*_.

Our results generally highlight a need for a better understanding of how physical processes in river channels drive river networks toward optimal configurations.

The framework we present has broad relevance to the study of branching networks and may be applied to better understand the formations of other geomorphic systems, such as deltaic and tidal networks. Geomorphic networks are dominated by mechanical processes and do not exhibit a comparable degree of three-dimensional complexity to superficially similar biological networks such as trees, neuron arbors, or vascular systems. Despite these clear differences, the framework we present in this study may conceivably be adapted to analyze any branching network in nature.

## 1. Conclusions

The geometry of the junction between two channels in a river network can be regarded as an optimization problem: what is the best way to arrange the links so as to minimize the hydraulic expenditure of energy? The equations that solve this optimization problem show that optimal junction geometries can be found given three key variables: the drainage area ratio of the confluent channels, the drainage area–discharge scaling exponent (*c*), and the drainage area–channel gradient scaling exponent (known as the concavity index, *θ*).

Two of these variables, the exponents *θ* and *c*, vary systematically with climate aridity (36, 44, 45), thereby theoretically linking junction geometry and climate aridity. This insight provides an analytical basis for the observed sensitivity of junction geometry to climate aridity—as reported in this study and others previously (34–36).

Our analysis reveals that network junctions with approximately equal tributary drainage areas (*A*_*R*_ ≈ 1) exhibit geometrically symmetrical junctions. This is consistent with the predictions of optimal junction theory. Junctions with asymmetric tributary drainage areas (*A*_*R*_ >  0.1) have asymmetric geometries, although these junctions do not exhibit perfect optimality. In other words, we find that relatively minor tributaries tend to join large rivers at the outside apex of large bends. This tendency has been reported in computational OCNs (14) suggesting such junction configurations may facilitate optimality at the scale of the entire network. In any case, this appears to be a fundamental structural characteristic of river networks.

The results from our global analysis of river network geometry are consistent with the theory that river networks evolve toward optimal planform geometries (6, 13, 14, 20–22) providing further evidence that branching networks in nature are unified by the principle of energetic optimization.

## Materials and Methods

The quality and spatial coverage of any junction angle dataset is dependent on the channel network from which it is derived. Previously published datasets have been hindered by limited spatial extent (35, 36) or the relatively coarse resolution of the input channel networks. Both Seybold et al. (35) and Getraer and Maloof (36) used the 30 m resolution NHDPlus V2 channel network covering the United States, while Seybold et al. (34) used the global 90 m resolution HydroSHEDS river network dataset (71).

Our global junction angle dataset was derived from a global channel network we extracted from NASA’s 30-m resolution void-filled Shuttle Radar Topography Mission Digital Elevation Model Version 3 (SRTM-DEM) (72). Initially, the SRTM-DATA were compiled into a DEM extending between 60°N and 56°S. High Arctic regions and Antarctica were excluded from analysis. Computational limitations demanded tiling this DEM prior to channel extraction. To limit the subdivision of basins, we used buffered basin outlines from the global HydroBASINS dataset (71) as “cookie-cutters” and clipped out a 30 m resolution SRTM-DEM tile for every individual basin in the HydroBASINS dataset larger than 10 km^2^ (for more information on buffering see *SI Appendix*). Based on centroid coordinates, each basin tile was projected into the relevant Universal Transverse Mercator (UTM) zone prior to hydrological processing. The largest of Earth’s drainage basins span several UTM zones, resulting in the distortion of drainage areas at the longitudinal extremities of the largest basins. To avoid this problem, we subdivided any basins with a drainage area greater than 200,000 km^2^. This was achieved by resampling the basin DEM tile to 250 m resolution (to facilitate efficient processing) and using the open-source topographic analysis software LSDTopoTools (73) to extract all complete subbasins with drainage areas between 10 and 200,000 km^2^. The output subbasins were then used as “cookie-cutters” to clip 30-m DEM basin tiles. These tiles were then buffered and processed alongside the input HydroBASINS basin outlines.

To prepare for hydrological flow routing, each basin DEM tile was preprocessed using the hybrid filling and carving algorithm of Lindsay (74). To avoid treating large internally drained basins as topographic depressions when “filling” the DEM, a hydrological sink was burned into the DEM at the location of the lowest point in every substantial internally drained basin. The sink locations, taken from a global dataset of endorheic basins (75), were originally derived from the HydroSHEDS global channel network dataset (71), with each sink manually verified.

Hydrological flow routing and channel extraction were then performed on every basin tile using LSDTopoTools (73). When extracting channel networks, channels were initiated at a threshold drainage area of 1 km^2^.

Once a channel network was generated, LSDTopoTools (73) was used to automate the measurement of every junction angle and the drainage area, length, and slope of every network link. The LSDTopoTools junction angle measurement tool works by performing orthogonal linear regression through all the points that together form a channel link. This is the same approach as Seybold et al. (35). Junction angles are then measured as the angles formed between the regression lines fitted to each of the three links meeting at any junction. We remove junctions where any of the three segments (e.g., two tributaries and a receiver segment) have fewer than eight pixels.

Artifacts in junction angle geometry can arise from the application of flow routing algorithms across relatively flat, low-gradient landscapes. To remove these artifacts from the dataset, any junctions composed of a channel segment with a gradient less than 0.0001 (1 m elevation loss in every 10 km) were omitted from analysis.

A global dataset of aridity (76) was used to assign every junction an aridity index (AI) value based on its location. The aridity index is the ratio of mean annual precipitation to mean annual evapotranspiration, with lower values indicating progressively more arid climates. To avoid the analysis of junctions where perhaps no real channels exist due to lack of any streamflow accumulation, all junctions located within “hyperarid” regions (aridity index < 0.03) were removed from the dataset. These regions are predominantly located in the deserts of North Africa, the Arabian Peninsula, and Central Asia (76). Please refer to *SI Appendix* for extended methods.

## Supplementary Material

Appendix 01 (PDF)

## Data Availability

[.csv data file] data have been deposited in [Environmental Information Data Centre] (https://datashare.ed.ac.uk/handle/10283/4767?show=full; https://doi.org/10.7488/ds/3781).
