# Supplementary material for: Explaining the climate sensitivity of junction geometry in global river networks
Source: Proc Natl Acad Sci U S A. 2022 Dec 5;119(50):e2211942119. doi: 10.1073/pnas.2211942119 (PMC9897458; doi:10.1073/pnas.2211942119)
Supplement: Supplementary file 1 — Appendix 01 (PDF) [file pnas.2211942119.sapp.pdf]

PNAS
www.pnas.org

Supplementary Information for

Explaining the climate sensitivity of junction geometry in global river networks

Callum M. Strong and Simon M. Mudd

Callum M. Strong.
E-mail: callum.strong@ed.ac.uk

This PDF file includes:

Supplementary text
Figs. S1 to S11 (not allowed for Brief Reports)
Table S1 (not allowed for Brief Reports)
SI References

Supporting Information Text

1. Junction angle extraction procedures

For a detailed summary of our junction angle datset extraction methods please refer to the Fig. S1. The software package we used to perform this analysis is freely available at https://github.com/LSDtopotools. The following sections contain details, discussion and justification of our extraction parameters choices.

A. Basin outline buffering procedure. We used HydroBASINS basin outlines as 'cookie-cutters' to clip out DEM tiles to hydrologically process from the Shuttle Radar Topography Mission Digital Elevation Model (SRTM-DEM). The HydroBASINS dataset was derived from a resampled (90 m resolution) version of the 30 m SRTM-DEM. As such we expect basin boundaries extracted from the 30 m SRTM-DEM to be generally aligned with the HydroBASINS basin boundaries. However, as HydroBASINS outlines are provided at a relatively coarse 15s ($\sim$ 450 m at the equator) resolution, there are spatial discrepancies between basin boundaries extracted from 30 m SRTM-DEM data and the HydroBASINS basin boundaries at local scales. To account for these discrepancies and to allow for the extraction of fully intact basins, we buffered the edges of each basin tile prior to hydrological processing. For tiles with areas less than 100 km^2 this buffer was fixed at 2 km. For tiles with areas between 100 km^2 and 15625 km^2 the buffer radius (R_{buffer}) dependant on the area of the tile (A_{tile}) as according to:

$$R_{buffer} = 0.25\sqrt{A_{tile}} \quad [1]$$

For tiles with areas greater than 15,625 km^2 R_{buffer} was fixed at 25 km. This buffering ensures that when flow routing was performed on the 30 m SRTM DEM basin tiles the great majority of basins have no truncation of basin headwaters at the tile margins. There are rare instances where spatial discrepancies between our extracted basin boundaries and the HydroBASINS basin boundaries result from differences are significant enough to extend beyond the buffered zone. Given these disagreements highlight significant discrepancies in flow routing paths, all junctions located on pixels draining from such areas were rejected from the dataset as a precaution against measuring incorrect drainage areas.

B. Selecting a channel extraction threshold. When extracting channel networks from topographic data the location of channel heads strongly controls the density and topology of the resulting network. While it is possible to apply algorithms to extract channel heads from topographic data (1) they require high pixel resolution and considerable processing time - constraints which made this approach unfeasible in our analysis. Instead we opted to use a simplistic threshold approach, whereby channels are initiated at a threshold drainage area.

What constitutes an appropriate drainage area threshold for channel initiation? Using a threshold that is too low may result in the extraction of false junctions where no channels exist. Using a threshold that is too high would result in fewer extracted junctions, sacrificing statistical confidence. The scale at which junction angles are measured is also controlled by the extraction threshold as channel links will be longer when this threshold is higher.

In 21 field studies of channel heads (2) the largest recorded initiation threshold area for a channel was 0.8 km^2 with the great majority of channels initiated below a threshold of 0.2 km^2. We opted to use a relatively conservative threshold for channel extraction of 1 km^2.

C. Hydrological flow routing. For surface flow-routing algorithms to properly extract drainage networks input topographic data should be free of voids and topographic depressions - these features behave as hydrological sinks, terminating extracted channels and falsely depriving downstream channels of drainage area. To avoid voids we used SRTM-DEM Version 3 (3) which is void-filled, having been patched with alternative data where voids in the original data existed. Although topographic depressions are not artefacts, it is standard practice when preparing DEM's for hydrological applications to assume flow into the depression has a hydrological connection to the surrounding watershed. DEM processing to maintain hydrologic connectivity can be accomplished by either filling depressions, breaching obstructions, or some combination of the two. For this study we used the hybrid breaching and filling algorithm of Lindsay (4) with the slope of filled surfaces set to 0.0001. Employed on the void-filled SRTM dataset, this algorithm results in a processed DEM wherein each pixel has a continuous downstream flow-path that terminates at the DEM margin. A weakness of this approach is that anywhere depressions are filled, planar low-gradient surfaces are artificially generated. This results in an artificial flow network pattern through filled pixels. We mitigate this effect in two ways: explicitly accounting for large internally drained basins and removing any junctions with a channel slope below the filled surface gradient (0.0001).

Filling errors are most problematic in internally drained (endorheic) basins. When fill algorithms are applied, large areas of completely artificial channel networks are generated. Due to the filling process these channels effectively spill over into neighboring basins causing drainage area errors to cascade downstream. Endorheic basins constitute approximately 18 percent of the Earth's land surface. To address the endorheic basin problem we generated an artificial hydrological sink at the location of the lowest elevation point within every significant internally drained basin, preventing the basin from being algorithmically filled. This was achieved using a global dataset of endorheic basins (5) derived from the HydroBASINS dataset (6).

In lowland regions DEM-based extraction of river networks may result in poor matches between the true location of channels and DEM-based channel locations due to filling and errors in flow routing in low relief terrain (7, 8). We mitigate these errors by rejecting junctions where any channel link has a gradient lower than a threshold, set to 0.0001.

Using the SRTM-DEM data for hydrological processing presents various other problems related to how the DEM was derived and what the data is capable of representing. SRTM-DEM is a digital surface model (DSM) which does not distinguish between

the real land surface and other natural/artificial surface features. Features such as urban developments, dense forests, and hydro-power/reservoir developments are represented as topography in the SRTM-DEM and these structures can result in the misrepresentation of real hydrology. Similarly, poor-quality flow routing can occur where rivers are confined in gorges narrower than the DEM pixel resolution (30 m) or where drainage does not occur at the land surface (e.g. karstic landscapes with subterranean rivers). No attempt was made to mitigate these effects in the channel network extraction and poorly represented channels may impact the location and geometry of some extracted junctions. The very large number of junction angles in the dataset (25,913,054) are assumed to statistically mitigate for any locally misrepresented hydrology.

D. Junction angle measurement. Once the DEM is preprocessed, including steps to avoid artefacts caused by endorheic basins (see above) and continuous flow paths are computed using the hybrid carving and filling algorithm (4), we use the resulting flow network to extract the junction network. Junctions each have a receiver junction (base level junctions' are their own receivers) and two or more donor junctions. We reject any junction that has more than two donor channels (this is extremely rare), and base level junctions. For each junction, we then add the location of every pixel along the channel pathway from the two donor junctions to the junction in question, and from that junction to the receiver junction. Base level junctions are ignored. The bearing of each of these three segments is calculated based on orthogonal regression (that is, for the set of channel pixels in each of the three segments, a line is fitted that minimises the sum of squared perpendicular distances from the data points to the regression line). These bearings are then used to calculate the junction and bending angles using standard vector calculations. The spacing between junctions is determined by the channel extraction threshold: smaller thresholds will have greater network density and thus more closely spaced junctions and shorter channel segments. We use channel segments between junctions rather than segments made up only of channel pixels near the junctions because we are interested in capturing the structure of how channels drain the entire landscape rather than focusing on the hydraulics near the confluence of tributaries. Any junction with a channel segment composed of less than 8 pixels is rejected. This avoids the inclusion of junctions dominated by local hydraulics and, more importantly, provides sufficient degrees of freedom for angular calculations from the linear regression to avoid the production of systematic values. As mentioned previously, we also eliminate junctions where any three of the confluent links have a gradient less than a threshold values to minimise errors from poorly located channels in low relief terrain. The criteria that a junction must include three segments with lengths greater than or equal to 8 pixels means that in large scale images of the channel network, such as an Figure 1, some locations that appear to have a junction are not added to the dataset. We illustrate examples of such conditions in Figure S2.

2. Optimal junction geometry across the full range of the energy scaling exponent

In the optimal junction model the parameter γ prescribes the relative cost weighting of channel links. When $\gamma = 1$ there is no energy-saving advantage for two links to join and form a junction, and hence no optimal junction angle solution. When $\gamma < 1$ there is a cost minimising advantage for the two links to join and form a junction. As γ decreases the optimal angle of α becomes wider and the optimal bending angles narrow as the energy-saving advantage of lengthening the resultant channel becomes progressively greater (Fig. S3). When $\gamma = 0$ all the links have the same weighting and finding the minimum energy configuration becomes a problem of total length minimisation, solved by all angles equalling 120° (Fig. S3). In optimal model junctions the value of γ predominantly controls α, with α narrowing as γ increases towards unity. The range of possible optimal junction angles is fundamentally constrained within the limits: $0° \leq \alpha \leq 120°$, $120° \leq \beta_1 \leq 180°$, and $90° \leq \beta_2 \leq 180°$.

3. Variability of the energy scaling exponent

Implicit in our calculated optimal junction geometries is the assumption that the energy scaling exponent γ is constant within all the links of a model junction.

Our results show that apparent values of γ decrease with increasing drainage area (as is consistent with hydrological evidence for a downstream decrease in c (9–15)). Therefore predictions made by employing the assumption of constant γ cannot be expected to perfectly match reality when the confluent tributaries are disparately sized. We mathematically investigated whether this assumption could be responsible for the discrepancies we observe between theory and reality.

Taking the case where the energy scaling exponent γ varies with drainage area, each link (i) with a unique drainage area value (A_i) will have a γ value independent of the other links' γ values such that the weighting of each link in Zamir's (16) optimal junction equations becomes $A_i^{\gamma_i}$:

$$\cos\alpha = \frac{A_0^{2(\gamma_0)} - A_1^{2(\gamma_1)} - A_2^{2(\gamma_2)}}{2A_1^{\gamma_1}A_2^{\gamma_2}} \qquad [2]$$

$$\cos(\beta_1) = \frac{A_2^{2(\gamma_2)} - A_0^{2(\gamma_0)} - A_1^{2(\gamma_1)}}{2A_0^{\gamma_0}A_1^{\gamma_1}} \qquad [3]$$

$$\cos(\beta_2) = \frac{A_1^{(2\gamma_1)} - A_0^{2(\gamma_0)} - A_2^{2(\gamma_2)}}{2A_0^{\gamma_0}A_2^{\gamma_2}} \qquad [4]$$

At a junction where two links (i_1 and i_2) join to form a resultant link (i_0), the drainage area is additive according to a simple bifurcation rule:

$$A_0 = A_1 + A_2 \tag{5}$$

In real rivers, A increases modestly from the upstream end of a link to the downstream end as areas proximal to the link drain into it. For simplicity we disregard these local increases in A and assume the A of any junction link conforms to Eq. 5. The drainage area ratio (A_R) of a junction is given by:

$$A_R = \frac{A_2}{A_1} \quad \text{where} \quad A_1 \geq A_2 \tag{6}$$

Using the bifurcation rule (5) and an A_R (6) the optimal junction equations (2, 3 and 4) can be simplified to (17):

$$\cos\alpha = \frac{(1+A_R)^{2(\gamma_0)} - 1 - A_R^{2(\gamma_2)}}{2A_R^{\gamma_2}} \tag{7}$$

$$\cos(\beta_1) = \frac{A_R^{2(\gamma_2)} - (1+A_R)^{2(\gamma_0)} - 1}{2(1+A_R)^{\gamma_0}} \tag{8}$$

$$\cos(\beta_2) = \frac{1 - (1+A_R)^{2(\gamma_0)} - A_R^{2(\gamma_2)}}{2A_R^{\gamma_2}(1+A_R)^{\gamma_0}} \tag{9}$$

When $A_R = 1$ we find:

$$\cos\alpha = \frac{2^{2\gamma_0} - 2}{2} \tag{10}$$

$$\cos(\beta_1) = \cos(\beta_2) = -2^{\gamma_0 - 1} \tag{11}$$

When $A_R = 1$ optimal junction geometry is sensitive only to γ_0, the γ value of the resultant channel. When $A_R \neq 1$, optimal junction geometry is sensitive to the γ values tributary and resultant channel links.

And when $A_R = 0$ we find analytically that optimal junction geometry becomes independent of γ with optimal angles of $\alpha = 90°$, $\beta_1 = 180°$, and $\beta_2 = 90°$. As the larger bending angle is predicted to approach 180° irrespective of the link γ values, the assumed constancy of γ cannot be producing the discrepancy between observed and predicted junction angles when the drainage area ratio approaches zero.

4. An alternative derivation of optimal junction angle equations using tributary slope ratio

The area ratio, A_R, is linked to the slope ratio, S_R, through the empirical slope-area scaling law (i.e., $S = k_s A^{-\theta}$). It is therefore theoretically possible to derive versions of Roy's (17) optimal junction angle equations (Eqs. 7, 8 and 9) formulated in terms of S_R:

$$\cos\alpha = \frac{(1+S_R^{\theta})^{2\gamma} - 1 - S_R^{\theta+2\gamma}}{2S_R^{\theta+\gamma}} \tag{12}$$

$$\cos\beta_1 = \frac{S_R^{\theta+2\gamma} - (1+S_R^{\theta})^{2\gamma} - 1}{2(1+S_R^{\theta})^{\gamma}} \tag{13}$$

$$\cos\beta_2 = \frac{1 - (1+S_R^{\theta})^{2\gamma} - S_R^{\theta+2\gamma}}{2S_R^{\theta+\gamma}(1+S_R^{\theta})^{\gamma}} \tag{14}$$

In the main body of this paper we have presented Roy's (17) original equations using A_R. We prefer the use of A_R for three reasons. Firstly, arriving at optimal predictions using a version of the optimal junction angle equations incorporating the slope ratio requires the assumption of a reference θ value to transform the bifurcation rule ($A_0 = A_1 + A_2$) so that S_0 can be formulated. Introducing this third variable parameter significantly complicates the process of finding optimal junction configurations. Secondly, drainage area (A) data from real rivers is much less noisy than gradient (S) data. Finally we believe A represents a more fundamental parameter than S in the context of network geometry as S is generally thought to be controlled by A through time varying erosion and deposition.

5. Does the 'fit' of straight lines to channel links vary with link gradient and link length?

When measuring junction geometry it is necessary to approximate each channel link in the channel network as a straight line. In our analysis this is achieved by performing an orthogonal linear regression through the points that compose each channel link. The sinuosity and curvature of real river channels varies, so the 'fit' of the vector line to the real channel link also varies. Does 'fit' vary systematically with factors such as the length and gradient of the channel link?

To analyse 'fit' the best metric available to us is the R^2 value of the lines fitted to the channel links. In effect the R^2 tells us how well each channel link can be approximated by a straight line. The R^2 therefore serves as a rough proxy for planform channel sinuosity, with high values representing straighter channel links and low values representing more sinuous channel links.

We plotted the R^2 value for all receiver channel links against link gradient (Fig. S9) and link length (Fig. S10). Receiver channel links were chosen so as to avoid duplication of links in the dataset as many receiver channel links subsequently form tributary channel links.

Median R^2 values increase (from a low of ~ 0.55) with increasing channel gradient up to a stable value of ~ 0.8 for links with gradients exceeding 0.02 (Fig. S9). Channel sinuosity is know to decrease with increasing channel slope (18, 19) likely driven by associated changes in channel confinement and sedimentary dynamics. For illustration, rivers with fine, cohesive sediment tend to meander, whereas steeper incising channels dominated by the bedload transport of coarse sediment tend to be confined by valley walls and flow more directly downslope (19). The variations of 'fit' with channel gradient that we observe in our dataset are therefore consistent with the known variation of river planform sinuosity with channel slope.

Median R^2 values decrease slightly from ~ 0.78 to ~ 0.72 as link length increases from 0 to 5 km (Fig. S10). The great majority of links in our dataset fall within this length range. For the rare links longer than 5 km R^2 values increase with length. For links of 20 km or more R^2 values are ~ 1.

Pertinent to our analysis of junction geometry, the R^2 values we observe in our dataset, although noisy, are in general reassuringly high. This demonstrates that, on average, the vectors we have fitted to channel links are reasonable approximations.

6. Verification of Roy's optimal geometry equations

We have verified Roy's (17) optimal geometry equations with a brute-fore iterative search for the optimal configurations, where 'optimal' is defined as the minimum possible value of energy expenditure (E) calculated by:

$$E \propto L_1 w_1 + L_2 w_2 + L_3 w_3 \quad [15]$$

Where w_i is the weighting factor of link (i). Calculating E for any junction configuration requires as input the starting point of each tributary segment (A and B), the end point of the resultant channel segment (C), the weighting factor for the tributary links and the angles α, β_1 and β_2. These geometric constraints define the location of the junction and allow the computation of channel segment lengths (L_1, L_2 and L_3). We wrote a program which performs an optimised iterative search through thousands of possible configurations and returns the configuration with the lowest possible E. Optimal configurations delivered by this program perfectly match the results of Roy's (17) optimal geometry equations. The model input parameters can be chosen so as to represent the geometry of any real-world junction formed by two adjoining channel segments. Note that optimal solutions show no dependency on the pre-defined spatial domain, except where the geometry of the fixed points inherently constrains the junction angle.

The method outlined in the previous paragraph requires tedious geometric calculations to calculate channel segment lengths L_1, L_2 and L_3 from the model input parameters. Please refer to Fig. S11. Points A, B and C form $\triangle ABC$ with sides a, b and c. If point A is defined by Cartesian coordinates x_A and y_A (and so on for points B and C) the length of the triangle sides can be calculated using Pythagorean theory:

$$a = \sqrt{(x_B - x_C)^2 + (y_B - y_C)^2} \quad [16]$$

$$b = \sqrt{(x_A - x_C)^2 + (y_A - y_C)^2} \quad [17]$$

$$c = \sqrt{(x_B - x_A)^2 + (y_B - y_A)^2} \quad [18]$$

A circle can be constructed which passes through the junction, J, point B and point C with center O_2. The line which passes through O_2 and bisects side a forms a right angle triangle with the line connecting O_2 and C. The inscribed angle theorem can be used to give us the other angles of this triangle, $180° - \beta_2$ and $\beta_2 - 180°$ (Fig.S11). This procedure can be repeated with respect to points A, J and C to form the circle O_1 and another right angle triangle with angles $180° - \beta_1$ and $\beta_1 - 180°$. Standard trigonometry gives us the radii (r_1 and r_2) of the circles (O_1 and O_2):

$$r_1 = \frac{a}{2\sin(\beta_1)} \quad [19]$$

$$r_2 = \frac{a}{2\sin(\beta_2)} \quad [20]$$

Using the rule of cosines the angle γ can be calculated from sides a, b and c:

$$\cos(\gamma) = \frac{a^2 + b^2 - c^2}{2ab} \quad [21]$$

And it follows that:

$$\angle O_1 C O_2 = \gamma + \beta_1 + \beta_2 - 180° \quad [22]$$

The line O_1O_2 bisects line L_3 and is therefore equal to twice the altitude of $\triangle O_1CO_2$ with respect to base O_1O_2. Finally, this allows the calculation of L_3:

$$L_3 = \frac{-2r_1 r_2 \sin(\beta_1 + \beta_2 + \gamma)}{\sqrt{r_1^2 + r_2^2 + 2r_1 r_2 \cos(\beta_1 + \beta_2 + \gamma)}} \quad [23]$$

Equations 19, 20, 22 and 23 can be combined to give an expression for L_3 in terms of sides a, b and c and junction angles β_1, β_2 and α:

$$L_3 = \frac{-2(\frac{a}{2\sin(\beta_1)})(\frac{b}{2\sin(\beta_2)})\sin(\beta_1+\beta_2+\arccos(\frac{a^2+b^2-c^2}{2ab}))}{\sqrt{(\frac{a}{2\sin(\beta_1)})^2+(\frac{b}{2\sin(\beta_2)})^2+2(\frac{a}{2\sin(\beta_1)})(\frac{b}{2\sin(\beta_2)})\cos(\beta_1+\beta_2+\arccos(\frac{a^2+b^2-c^2}{2ab}))}} \quad [24]$$

This last step can be repeated to also find L_1 and L_2.

Table S1. Climate classification for different Aridity Index values (20)

Aridity Index Value	Climate Class
< 0.03	Hyper Arid
0.03 – 0.2	Arid
0.2 – 0.5	Semi-Arid
0.5 – 0.65	Dry sub-humid
> 0.65	Humid

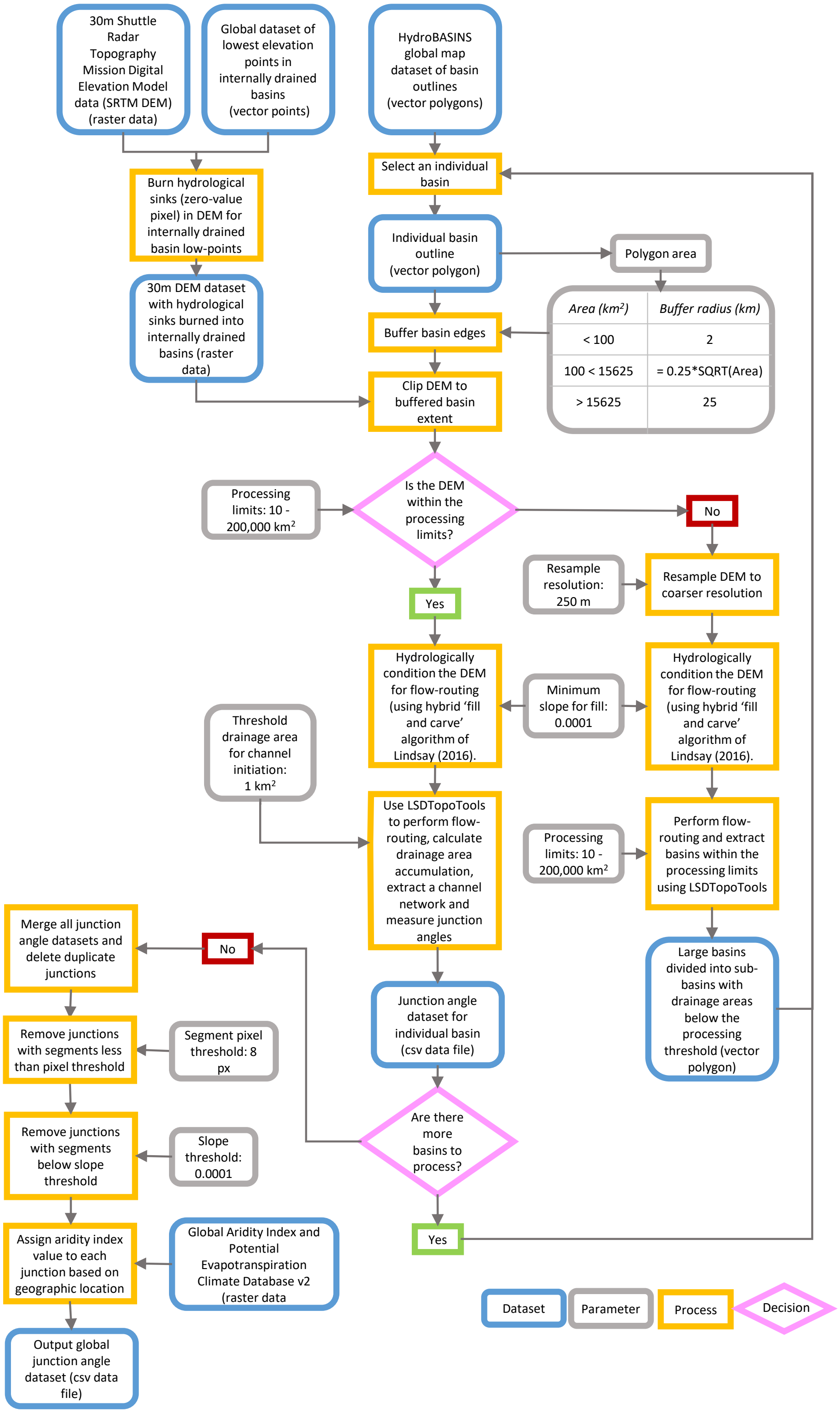

Fig. S1. Workflow for junction angle extraction and extraction parameters

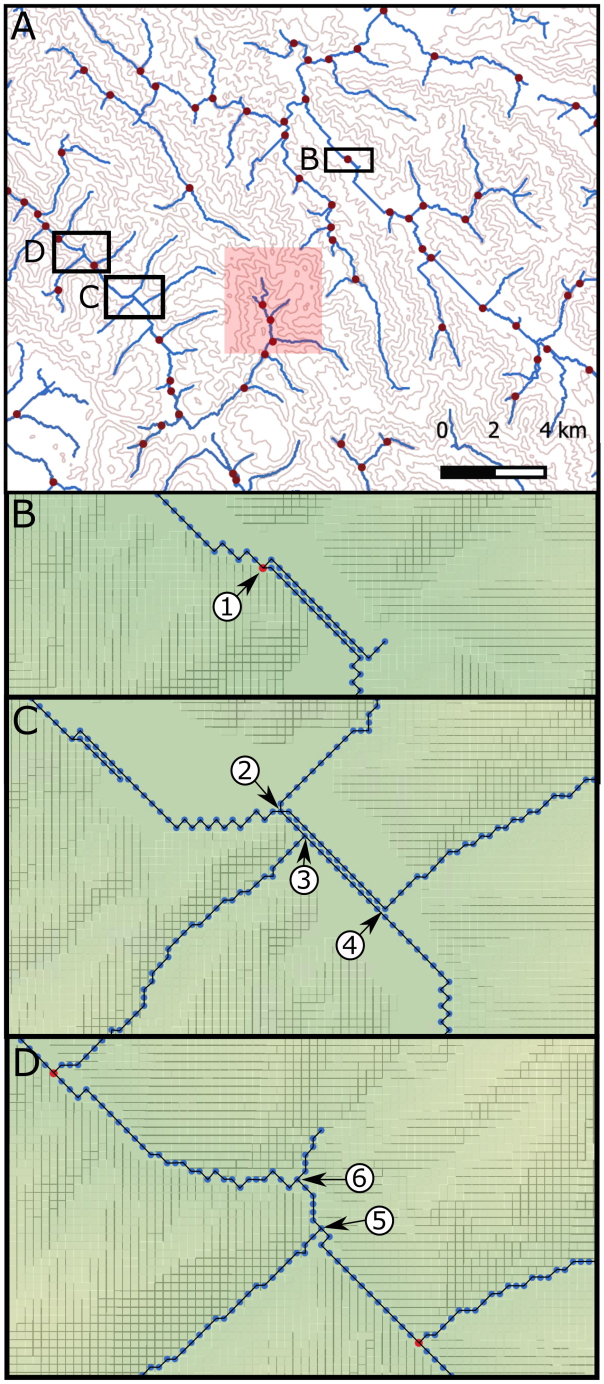

Fig. S2. Examples of complexities in extraction of junctions. We provide examples of junction selection or absence that can be obscured when looking at the dataset at regional scales. The junctions from this image have not yet had the gradient threshold applied (junctions where any one of the three segments has a gradient < 0.0001 is removed) but junctions with short segments (8 pixels or less) are removed. Panel A shows the same area as in Figure 1 in the main text. Panel B shows a junction (1) where all three segments are greater than 8 pixels but because of flat terrain one segment runs parallel to another and this parallel segment cannot be seen at the scale of panel A. Such junctions are frequently eliminated by the gradient threshold. Panel C appears to have 3 junctions but two of these are removed (junctions 2 and 3) because both have a segment shorter than 8 pixels. The other apparent junction (4) is actually not a true junction due to parallel flow routing - once again this detail is not visible at the scale of Panel A. In panel D both junctions (5 and 6) are also removed due to short link lengths.

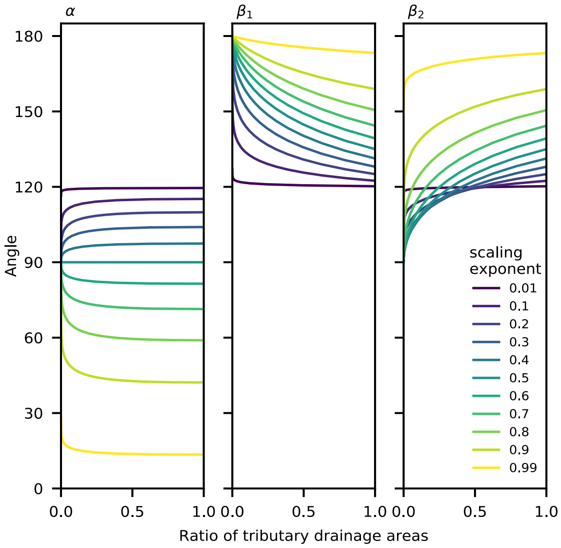

Fig. S3. Optimal junction geometries generated using Eqs. 2, 3 and 4 for selected values of the energy scaling exponent γ and across all possible values of the tributary drainage area ratio.

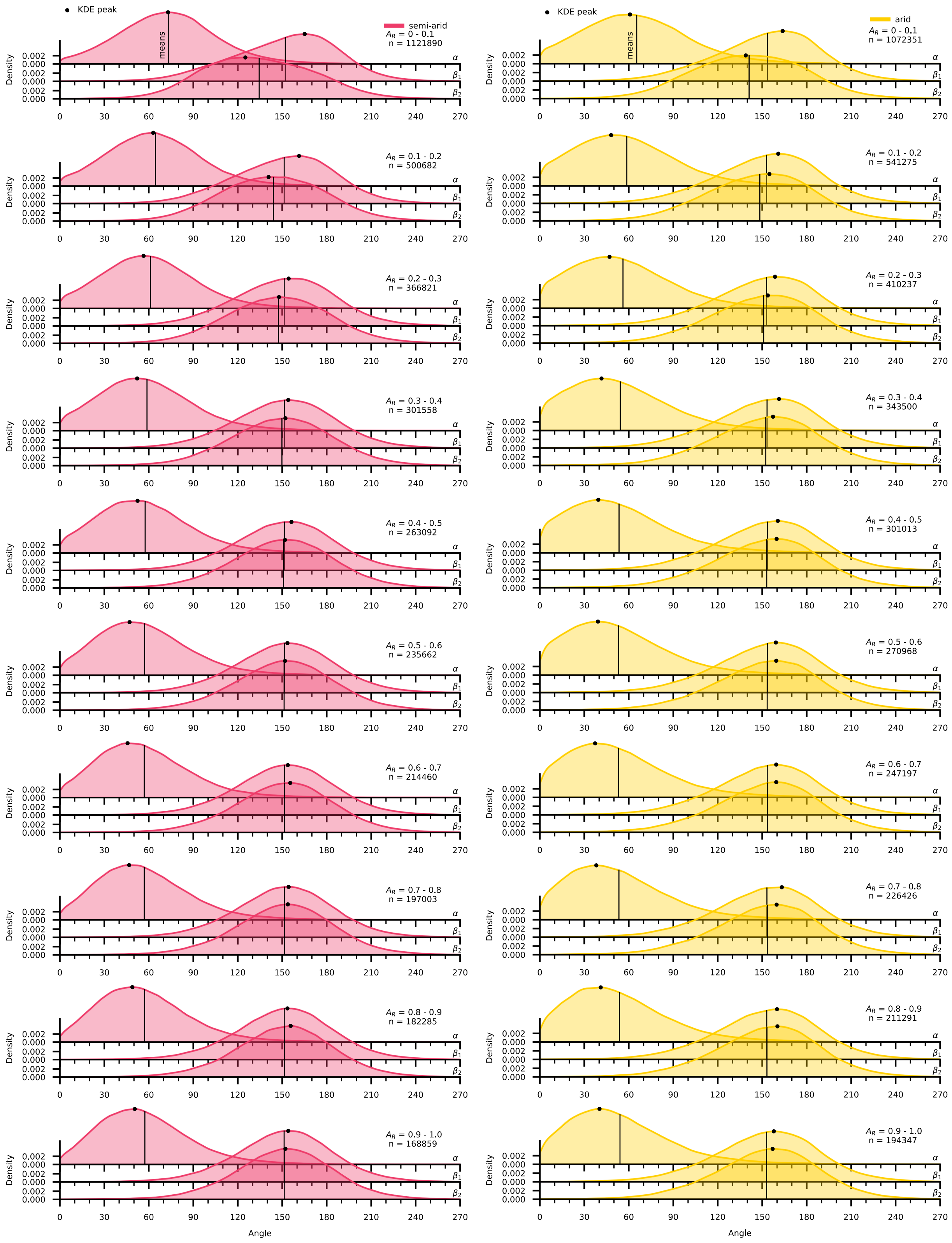

Fig. S4. Kernel density estimate distributions for arid and semi-arid junctions binned by tributary drainage area ratio. Black vertical lines are mean values and black dots are the KDE peak values.

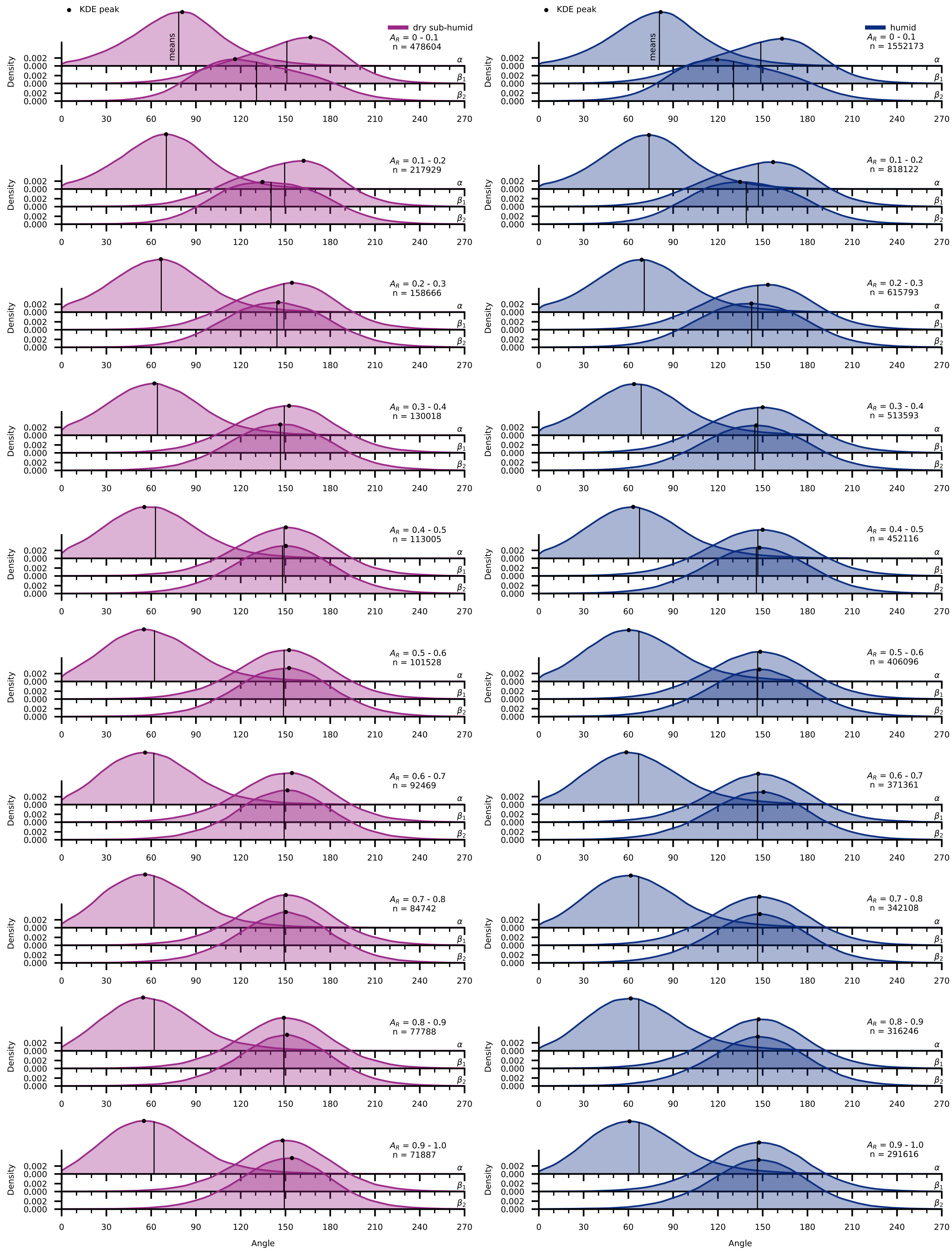

Fig. S5. Kernel density estimate distributions for humid and dry sub-humid junctions binned by tributary drainage area ratio. Black vertical lines are mean values and black dots are the KDE peak values.

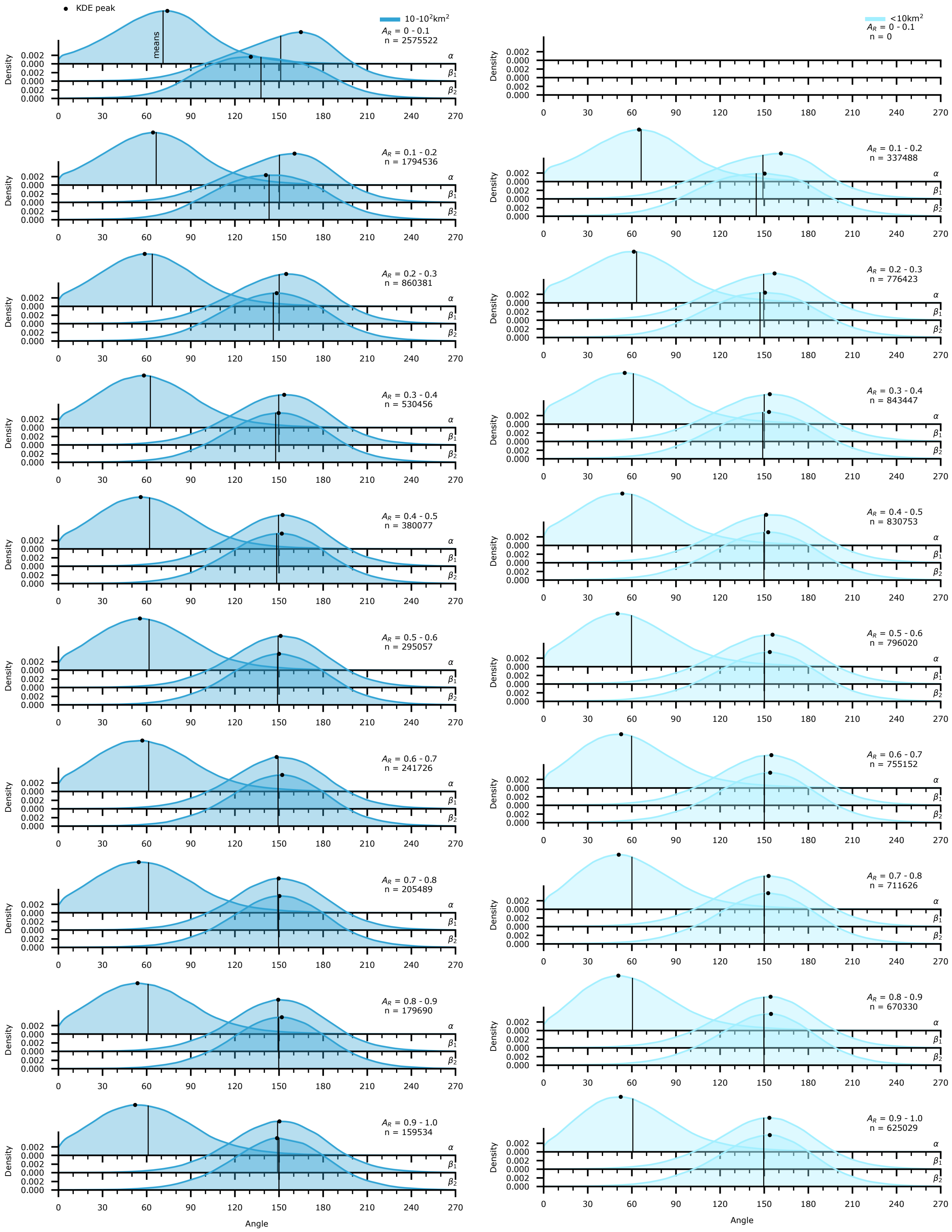

Fig. S6. Kernel density estimate distributions for junctions with total drainage area less than $10km^2$ (left), and total drainage area between $10km^2$ and $100km^2$ (right) binned by tributary drainage area ratio. Black vertical lines are mean values and black dots are the KDE peak values.

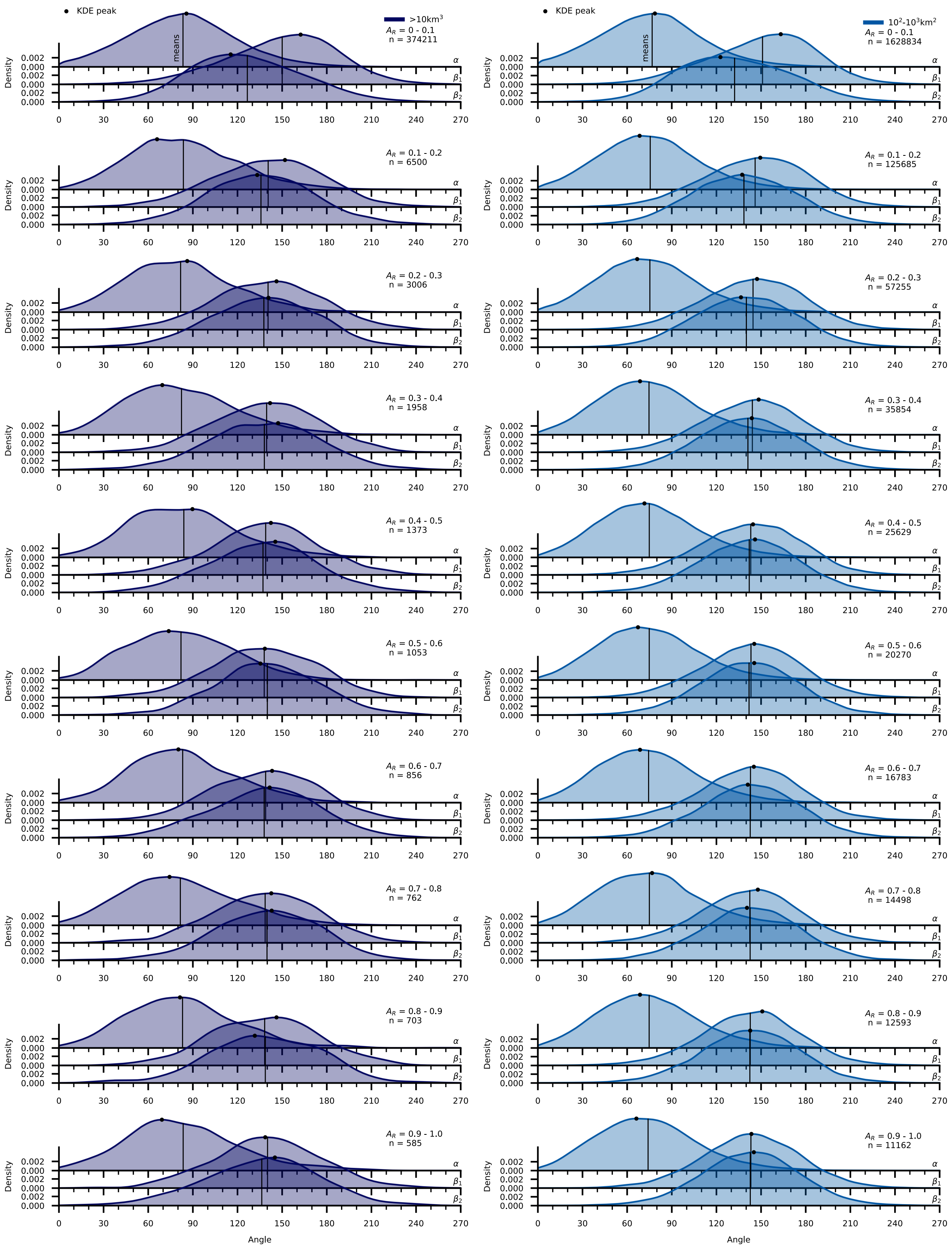

Fig. S7. Kernel density estimate distributions for junctions with total drainage area between $100km^2$ and $1000km^2$ (left), and total drainage area greater than $1000km^2$ (right) binned by tributary drainage area ratio. Black vertical lines are mean values and black dots are the KDE peak values.

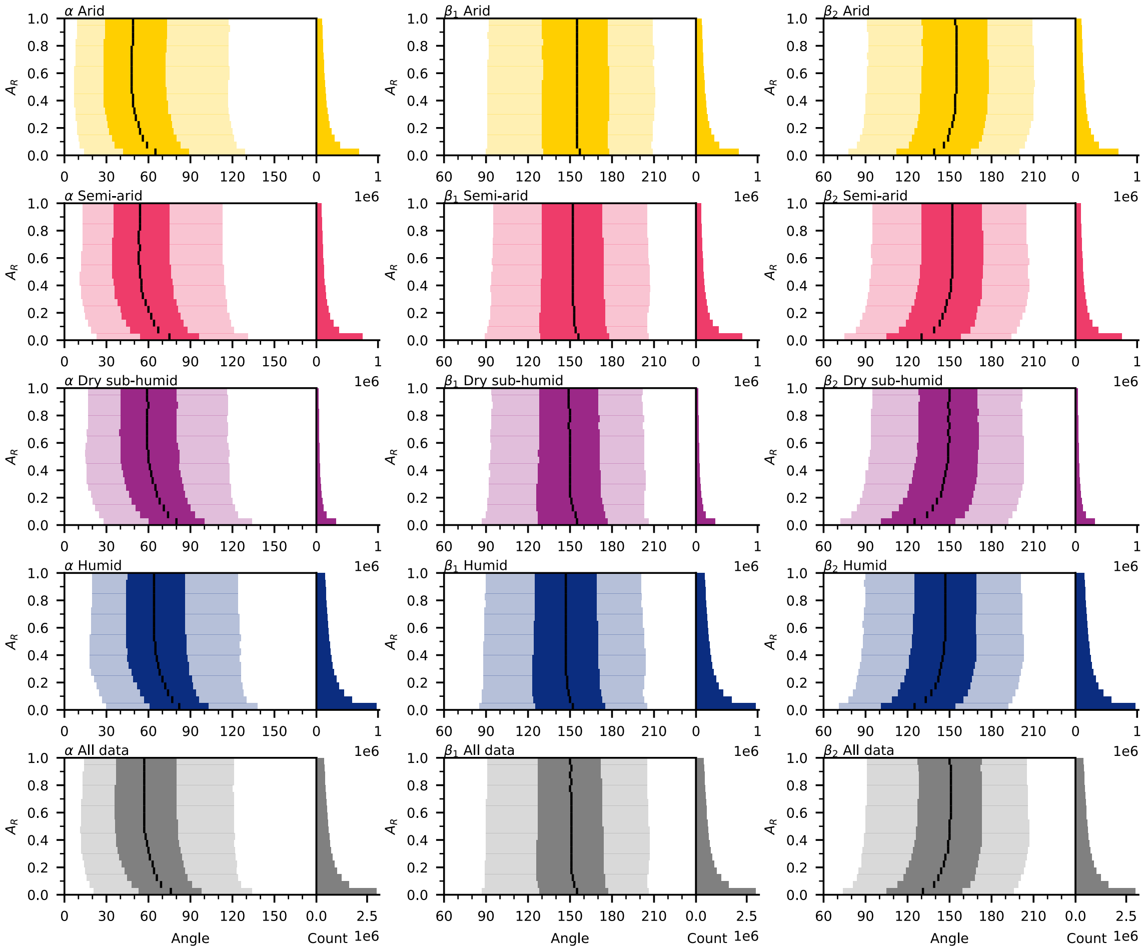

Fig. S8. Junction angles (α) and bending angles (β_1, β_2) sorted by climate aridity zone and binned by drainage area ratio (A_R). Black line is the median value, the darker coloured shading represents the 25th to 75th data percentiles, and the lighter coloured shading represents the 5th to 95th data percentiles. The histogram along the right hand side of the plots is a count of the number of junctions in each of the 20 bins.

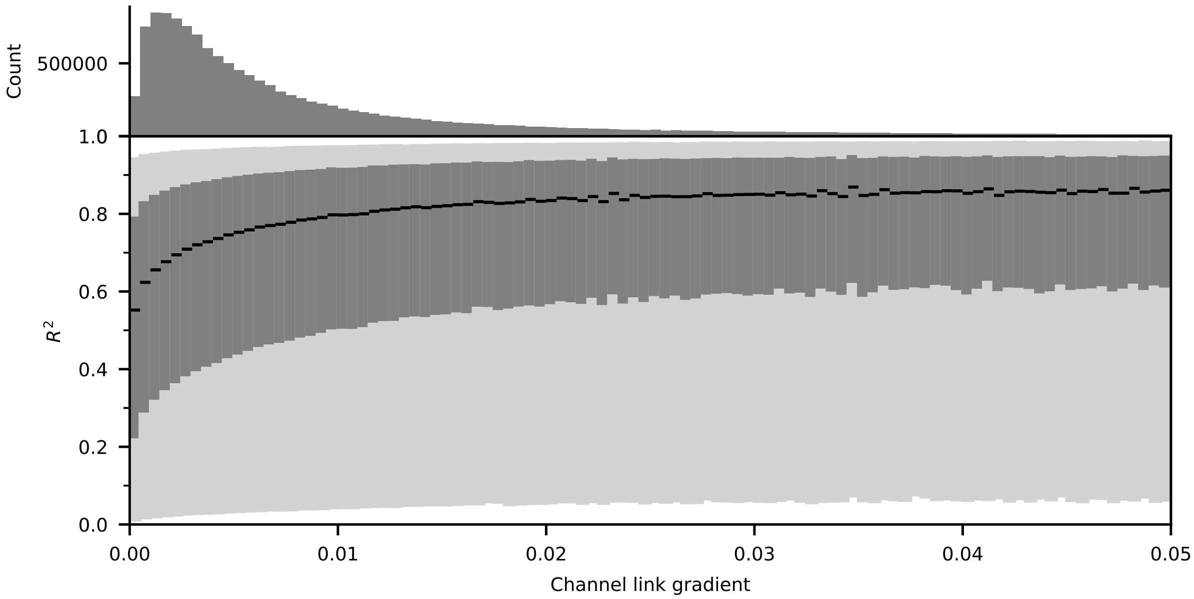

Fig. S9. This plot shows the relationship of the R^2 value from the orthogonal linear regression performed on each channel link (effecively a measure of 'fit') and the average link gradient. Black line is the median value of each data bin, the darker grey shading represents the 25th to 75th data percentiles, and the lighter grey shading represents the 5th to 95th data percentiles. The histogram at the top of the plot is a count of the number of channel links in each bin.

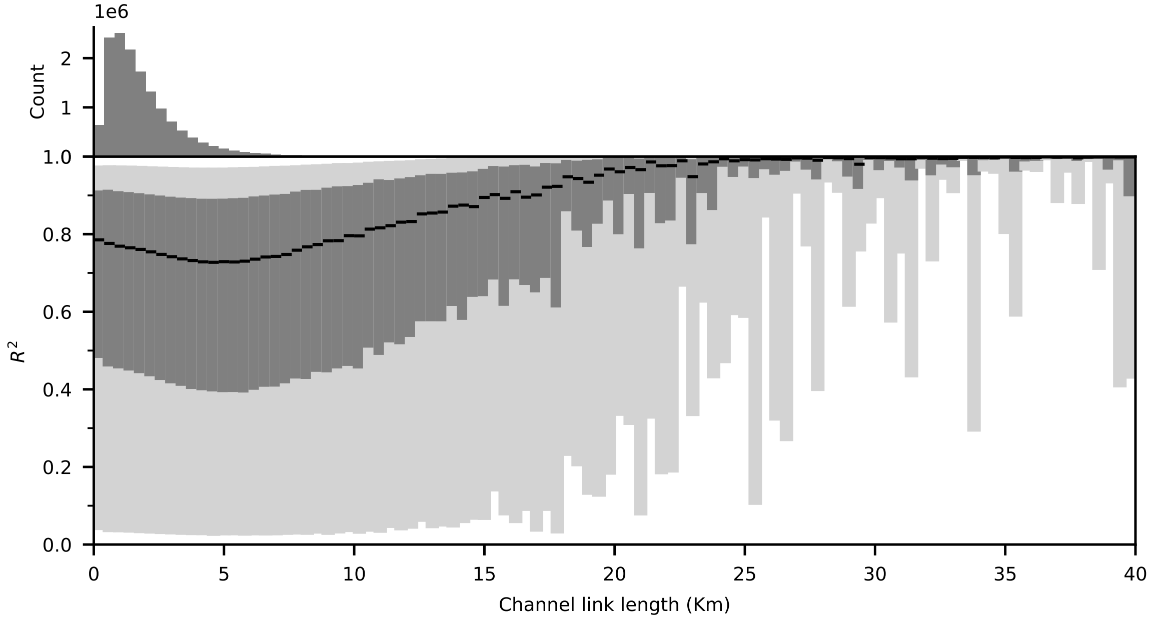

Fig. S10. This plot shows the relationship between the R^2 value from the orthogonal linear regression performed on each channel link (effecively a measure of 'fit') and the link length. Black line is the median value, the darker grey shading represents the 25th to 75th data percentiles, and the lighter grey shading represents the 5th to 95th data percentiles. The histogram at the top of the plot is a count of the number of channel links in each bin.

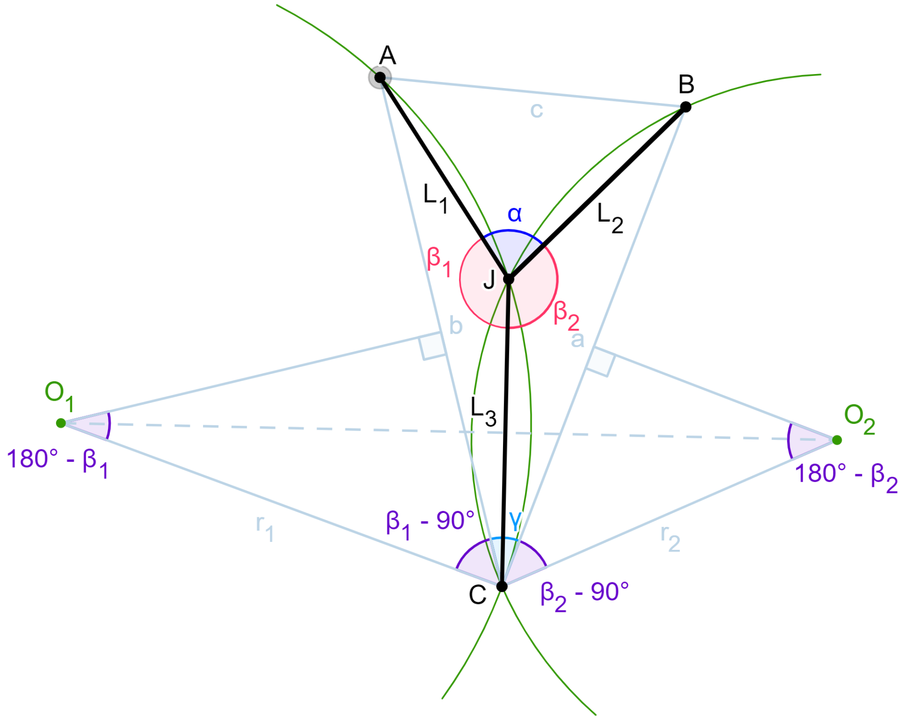

Fig. S11. Diagram of geometric junction angle model

References

1. FJ Clubb, SM Mudd, DT Milodowski, MD Hurst, LJ Slater, Objective extraction of channel heads from high-resolution topographic data. *Water Resour. Res.* **50**, 4283–4304 (2014) _eprint: https://agupubs.onlinelibrary.wiley.com/doi/pdf/10.1002/2013WR015167.
2. E Wohl, The challenges of channel heads. *Earth-Science Rev.* **185**, 649–664 (2018).
3. NASA Shuttle Radar Topography Mission (SRTM). Shuttle Radar Topography Mission (SRTM) Global. Distributed by OpenTopography. Accessed: 2021-01-13 (2013).
4. JB Lindsay, Efficient hybrid breaching-filling sink removal methods for flow path enforcement in digital elevation models. *Hydrol. Process.* **30**, 846–857 (2016).
5. JS Lee, Global modelling of connections between endorheic and exorheic basins (2018) Type: data set.
6. B Lehner, G Grill, Global river hydrography and network routing: baseline data and new approaches to study the world's large river systems. *Hydrol. Process.* **27**, 2171–2186 (2013) _eprint: https://onlinelibrary.wiley.com/doi/pdf/10.1002/hyp.9740.
7. D Yamazaki, et al., MERIT Hydro: A High-Resolution Global Hydrography Map Based on Latest Topography Dataset. *Water Resour. Res.* **55**, 5053–5073 (2019) _eprint: https://agupubs.onlinelibrary.wiley.com/doi/pdf/10.1029/2019WR024873.
8. Z Wang, et al., Basin-scale high-resolution extraction of drainage networks using 10-m Sentinel-2 imagery. *Remote. Sens. Environ.* **255**, 112281 (2021).
9. DC Goodrich, et al., Linearity of basin response as a function of scale in a semiarid watershed. *Water Resour. Res.* **33**, 2951–2965 (1997).
10. TB Ayalew, WF Krajewski, R Mantilla, Connecting the power-law scaling structure of peak-discharges to spatially variable rainfall and catchment physical properties. *Adv. Water Resour.* **71**, 32–43 (2014).
11. PV Mandapaka, WF Krajewski, R Mantilla, VK Gupta, Dissecting the effect of rainfall variability on the statistical structure of peak flows. *Adv. Water Resour.* **32**, 1508–1525 (2009).
12. ME Wilkinson, JC Bathurst, A multi-scale nested experiment for understanding flood wave generation across four orders of magnitude of catchment area. *Hydrol. Res.* **49**, 597–615 (2017).
13. RA DiBiase, KX Whipple, The influence of erosion thresholds and runoff variability on the relationships among topography, climate, and erosion rate. *J. Geophys. Res. Earth Surf.* **116** (2011) _eprint: https://onlinelibrary.wiley.com/doi/pdf/10.1029/2011JF002095.
14. KT Lee, NC Chen, BI Gartsman, Impact of stream network structure on the transition break of peak flows. *J. Hydrol.* **367**, 283–292 (2009).
15. KT Lee, JK Huang, Influence of storm magnitude and watershed size on runoff nonlinearity. *J. Earth Syst. Sci.* **125**, 777–794 (2016).
16. M Zamir, Optimality principles in arterial branching. *J. Theor. Biol.* **62**, 227–251 (1976).
17. AG Roy, Optimal Angular Geometry Models of River Branching. *Geogr. Analysis* **15**, 87–96 (1983) _eprint: https://onlinelibrary.wiley.com/doi/pdf/10.1111/j.1538-4632.1983.tb00771.x.
18. RPdM Frasson, et al., Global relationships between river width, slope, catchment area, meander wavelength, sinuosity, and discharge. *Geophys. Res. Lett.* **46**, 3252–3262 (2019).
19. SA Schumm, Sinuosity of alluvial rivers on the great plains. *Geol. Soc. Am. Bull.* **74**, 1089–1100 (1963).
20. A Trabucco, R Zomer, Global Aridity Index and Potential Evapotranspiration (ET0) Climate Database v2, (figshare), Fileset (2019).
